# Letter to the editor: Serum amylase on postoperative day one is a strong predictor of pancreatic fistula after pancreaticoduodenectomy: a retrospective cohort

**DOI:** 10.55730/1300-0144.5799

**Published:** 2023-12-04

**Authors:** Cengiz CEYLAN

**Affiliations:** Department of Surgery, Inönü University, Malatya, Turkiye

I have read the original article entitled “Serum amylase on postoperative day one is a strong predictor of pancreatic fistula after pancreaticoduodenectomy: a retrospective cohort” by Özşay O. et al [1] published in Turkish Journal of Medical Sciences (2023; 53 (5): 1271–1280). I want to congratulate the authors for this successful original article, and make some contributions. Postoperative pancreatic fistula (POPF) has been defined by the International Study Group of Pancreatic Surgery (ISGPF) [2]. Additionally, although various risk factors for POPF have been described in the literature, there is still no consensus [3]. The author emphasizes the elevated serum amylase on the first postoperative day as a strong predictor for POPF. However, I would like to highlight several important points that need attention.

Initially, postoperative hyperamylasemia, one of the diagnostic criteria for postpancreatectomy acute pancreatitis (PAPP), was defined in the ISGPF in 2021 [4]. The presence of PAPP is emphasized to be nonnegligible. Moreover, there are publications indicating that this condition can progress to total pancreatectomies following necrosis in the remnant pancreas [5–7]. Therefore, PAPP is regarded as a major complication after pancreatectomies. Although postoperative hyperamylasemia has been observed to increase in both postoperative pancreatic fistulas (POPF) and PAPP, it has been shown that medical drugs used during the perioperative period, such as ondansetron, ceftriaxone, propofol, clarithromycin, acetaminophen, can lead to pancreatitis and consequently induce hyperamylasemia [8].
